# Efficacy and Safety Assessment of Intrathoracic Perfusion Chemotherapy Combined with immunological factor Interleukin-2 in the Treatment of Advanced Non-Small Cell Lung Cancer: A Retrospective Cohort Study

**DOI:** 10.7150/jca.92624

**Published:** 2024-02-17

**Authors:** Qiang Cao, Jinyi Zhu, Xinyan Wu, Jiapeng Li, Yuquan Chen, Yanwei You, Xiaochen Li, Xufeng Huang, Yujie Zhang, Rizhu Li, Dan Han

**Affiliations:** 1 Department of Pharmacy, Nanjing Drum Tower Hospital, Affiliated Hospital of Medical School, Nanjing University, Nanjing, China.; 2 School of Medicine, Macau University of Science and Technology, 999078, Macau, Macao.; 3 Department of Earth Sciences, Kunming University of Science and Technology, 650093, Kunming, China.; 4 Affiliated Hospital of Weifang Medical University, School of Clinical Medicine, Weifang Medical University, Weifang, China.; 5 College of Veterinary Medicine, Sichuan Agricultural University, 610000, Chengdu, China.; 6 Undergraduate Department, University of Toronto, M2J4A6, Toronto, Canada.; 7 Institute of Medical Information/Library, Chinese Academy of Medical Sciences, 100020 Beijing, China.; 8 Division of Sports Science & Physical Education, Tsinghua University, Beijing 100084, China.; 9 The Third Affiliated Hospital of Shandong First Medical University, Jinan, 250000, Shandong, China.; 10 Faculty of Dentistry, University of Debrecen, Debrecen, Hungary.; 11 College of Agriculture, Henan University of Science and Technology, 471023, Luoyang, China.; 12 Department of Cardiothoracic Surgery, the Affiliated Hospital of Youjiang Medical University for Nationalities, 18 zhongshan 2nd Road, Baise, Guangxi Province, China.

**Keywords:** Interleukin-2 Treatment, immunotherapy, Advanced Non-Small Cell Lung Cancer, Intrathoracic Perfusion Chemotherapy, Efficacy and Safety Evaluation, Retrospective Cohort Study

## Abstract

**Objective:** This study evaluated the efficacy and safety of the gemcitabine and oxaliplatin intrathoracic perfusion chemotherapy (IPCGOR) regimen combined with interleukin-2 (IL-2) for advanced non-small cell lung cancer (NSCLC).

**Methods:** We conducted a retrospective analysis of 460 advanced NSCLC patients from the Yunnan Province Early Cancer Diagnosis and Treatment Project (June 2020-October 2022), assessing the IPCGOR and IL-2 combination. Outcomes were measured based on RECIST 1.1 criteria, focusing on objective response rate (ORR), disease control rate (DCR), median progression-free survival (mPFS), median overall survival (MOS), and treatment safety.

**Results:** The treatment demonstrated an ORR of 67.4%, a DCR of 97.4%, an mPFS of 8.5 months, and an MOS of 12.5 months. 14 patients underwent successful surgery post-treatment. Common adverse reactions were manageable, with no treatment-related deaths reported.

**Conclusion:** The IPCGOR combined with IL-2 regimen shows promising efficacy and a tolerable safety profile for advanced NSCLC. These findings suggest its potential as a reference for treating advanced NSCLC. However, the study's retrospective nature and single-center design pose limitations. Future research should focus on prospective studies, randomized controlled trials, and long-term outcome assessments, particularly in diverse patient subgroups, to further validate and refine the clinical application of this regimen.

## 1. Introduction

Lung cancer remains the most prevalent and deadly malignant tumor worldwide. It is the leading cause of cancer death among men and ranks second in incidence and mortality among women 1-3. Annually, lung cancer is responsible for 1.37 million deaths globally, constituting 18% of all cancer fatalities 4-5. Among lung cancer types, non-small cell lung cancer (NSCLC) accounts for about 85% of cases. NSCLC often goes undetected until advanced stages, limiting curative options like surgery or ablation 6-8.

Recent years have seen remarkable advancements in both local and systemic NSCLC treatments, significantly improving survival in advanced stages 9-10. Notably, there has been increasing focus on integrating chemotherapy with immunotherapy. Recent studies indicate that combining cytotoxic drugs with immune modulators can enhance treatment efficacy and patient outcomes in NSCLC 11-12. This approach seeks to leverage the direct tumor-killing effects of chemotherapy while harnessing the body's immune response to target cancer cells more effectively.

In this context, the gemcitabine and oxaliplatin intrathoracic perfusion chemotherapy (IPCGOR) has emerged as a promising treatment. IPCGOR delivers high concentrations of cytotoxic agents directly to the thoracic region, maximizing their tumor-killing potential 13-14. Traditional perfusion regimens like oxaliplatin plus thymosin (IIOT) require extended treatment times of about 60 hours, leading to discomfort and increased risks such as tube dislodgement and infections. In contrast, the IPCGOR regimen requires only 4 hours, markedly reducing treatment duration while maintaining effectiveness. This shorter regimen improves patient compliance and minimizes discomfort, representing a significant advancement in NSCLC treatment.

The combination of IPCGOR with cytokine drugs significantly improves tumor response rates and patient survival rates compared to IPCGOR alone. Interleukin-2 (IL-2), a pivotal immune molecule, plays a crucial role in this treatment regimen 15-16. As a cytokine, IL-2 is integral in regulating the activities of white blood cells that are responsible for immunity, such as T cells and natural killer cells. Its administration in combination with IPCGOR aims to boost the body's immune response against tumor cells 17-18. IL-2 stimulates the proliferation of activated lymphocytes, enhancing the body's capacity to identify and combat cancer cells 19-20. Moreover, it assists in mitigating the bone marrow suppression often caused by chemotherapy agents, thereby sustaining the patient's immune competence during treatment 21-22. The inclusion of IL-2 in this treatment regimen is based on the reason of that an activated immune system can significantly augment the efficacy of direct cytotoxic effects on tumor cells, offering a dual approach to combating advanced NSCLC 23-24. Raeber *et al.*'s study 25 shown that the combination of IPCGOR with IL-2 can activate Teff cells, promote immune activation, and reduce bone marrow suppression caused by IPCGOR, thereby enhancing anti-tumor effects.

Current, clinical investigations into the efficacy of IPCGOR in conjunction with IL-2 for the treatment of advanced NSCLC are notably limited. This research endeavors to elucidate the therapeutic efficacy and safety profile of IPCGOR paired with IL-2 in the management of advanced NSCLC by undertaking a comprehensive clinical retrospective cohort study with a substantial sample size.

## 2. Subjects and Methods

### 2.1 Subjects

Through the Yunnan Province Early Cancer Diagnosis and Treatment Project cohort, a retrospective analysis was conducted on 460 patients with advanced NSCLC from Yunnan Province, China, who underwent IPCGOR combined with IL-2 treatment from June 2020 to October 2022. Their clinical data were collected.

### 2.2 Study Population Inclusion Methods

#### 2.2.1 Case Selection and Exclusion Criteria

**Inclusion Criteria:**

(1) Pathologically confirmed diagnosis of advanced non-small cell lung cancer (NSCLC).

(2) Age range of patients: 18-65 years.

(3) Presence of at least one measurable lesion in the lung or mediastinum, as defined by RECIST 1.1 criteria.

(4) Life expectancy of more than 12 months, as estimated by the treating physician.

(5) Undergone treatment with the IPCGOR regimen combined with IL-2, and completion of at least one full treatment cycle. Additionally, the patient must have undergone a minimum of three tumor assessments following the treatment initiation.

(6) Provided voluntary informed consent, confirmed through a signed consent form.

**Exclusion Criteria:**

(1) History of other primary malignant tumors.

(2) Incomplete or insufficient clinical data for analysis.

(3) Presence of severe comorbidities, including but not limited to uncontrolled diabetes, severe renal dysfunction, active infections, or cardiovascular diseases that could compromise patient safety or study results.

(4) Concurrent use of other anti-tumor therapies or participation in other clinical trials during the study period.

(5) Refusal or inability to provide informed consent for participation in the study.

Patient selection and treatment administration were conducted under strict adherence to ethical guidelines. This study was conducted with the approval of the Ethics Committee of Kunming University of Science and Technology, with approval number KMUST202109327142.

#### 2.2.2 Treatment Regimen

IPCGOR infusion protocol: All patients underwent thoracentesis to remove pleural effusion. When the effusion volume was <200ml/d, a chest X-ray was repeated. After confirming good lung re-expansion, oxaliplatin 150 mg/m2 (2∼4 h) was injected through the catheter; gemcitabine 500mg/m2, once every 2 weeks.

IL-2 administration protocol: 1,000,000 IU/time, intravenous drip, twice a week.

#### 2.2.3 Observation Indicators and Evaluation Criteria

Routine blood tests, liver and kidney function, coagulation, alpha-fetoprotein (AFP) and other indicators were reviewed every 3 weeks. Enhanced upper abdominal computed tomography (CT) / magnetic resonance imaging (MRI) was reviewed every 6-8 weeks.

(1). Therapeutic efficacy was assessed based on the RECIST 1.1 criteria for solid tumors:

1. Complete Response (CR): Disappearance of all target lesions;

2. Partial Response (PR): A diminution of no less than 30% in the aggregate diameters of the designated target lesions, relative to the baseline measurements.

3. Progressive Disease (PD): An escalation in the sum of diameters of target lesions by a minimum of 20%, juxtaposed with the smallest cumulative measurement ascertained during the study, or the emergence of new lesions.

4. Stable Disease (SD): Neither sufficient shrinkage to qualify for PR nor sufficient increase to qualify for PD.

(2). Progression-Free Survival (PFS): PFS is delineated as the interval commencing with the initiation of therapeutic intervention and extending to either the observed advancement of the neoplasm or death attributable to any etiology.

(3). Overall Survival (OS) is defined as the time from the start of treatment to death from any cause.

(4). Adverse reaction evaluation criteria: According to the Common Terminology Criteria for Adverse Events (CTCAE 5.0), they are classified into grades 1-5.

### 2.3 Statistical Analysis

In this study, the statistical analyses were conducted using R software, version 4.0, chosen for its robustness and versatility in handling complex biomedical data. We determined the sample size based on preliminary data, aiming to achieve adequate power to detect significant differences in survival outcomes. The sample size calculation was based on anticipated PFS differences between treatment groups, ensuring at least 80% power to detect a clinically meaningful difference with an alpha level of 0.05. Quantitative data conforming to a normal distribution were expressed as the mean ± standard deviation. For data not following normal distribution, we reported median values, along with their range from minimum to maximum values. Categorical data were presented as frequencies and corresponding percentages. The survival analysis for Progression-Free Survival (PFS) and Overall Survival (OS) was conducted using the Kaplan-Meier estimator, chosen for its ability to effectively handle censored data and provide a visual representation of survival probabilities over time.A univariate analysis utilizing Cox proportional hazards regression was performed to identify potential predictors of PFS. This method was selected due to its capability to handle time-to-event data and adjust for potential confounding variables. After identifying statistically significant factors in the univariate analysis, we constructed a multivariate Cox proportional hazards regression model. This model was used to determine hazard ratios (HR) for PFS, along with their 95% confidence intervals (CI), providing a more comprehensive understanding of the factors influencing patient outcomes.The threshold for statistical significance was set at an alpha level of 0.01. A P-value of less than 0.01 was considered indicative of statistical significance, aligning with stringent criteria to minimize the likelihood of Type I error in our findings.

## 3. Results

### 3.1 Baseline Information

As shown in Table 1, a total of 460 patients were included in this study, comprising 409 males (88.91%) and 51 females (11.09%), with an average age of 51.3±9.8 years. 427 patients (92.83%) were in BCLC stage C, and 33 patients (7.17%) were in stage B. Among them, 241 patients (52.39%) experienced extra-pulmonary metastasis. Baseline AFP levels were ≥400 ng/mL in 339 patients (73.7%). The triplet therapy was the first-line treatment for 289 patients (62.8%), while the remaining 171 patients (37.1%) primarily underwent TACE, ablation, or received this regimen as a second-line or subsequent treatment after tumor progression. The median number of times patients received IPCGOR combined with IL-2 treatment was 3 (range 2-6).

### 3.2 Evaluation of the Efficacy of IPCGOR Combined with IL-2 Treatment in NSCLC

As shown in Table 2, as of October 2022, 310 patients (67.4%) achieved PR, 138 patients (30.0%) achieved SD, and 12 patients (2.6%) experienced progression. The ORR was 67.4%, and the DCR reached 97.4%. Among them, 14 patients experienced sufficient tumor reduction to be candidates for surgical intervention and have achieved CR following surgical resection to date. The median Progression-Free Survival (mPFS) was 8.5 months (95% CI: 6.1-10.2), and the Median Overall Survival (MOS) was 12.5 months (95% CI: 11.4-17.6). In patients receiving first-line treatment, the ORR was 80.42%, and DCR reached 98.94%. When IPCGOR combined with IL-2 treatment was used as second-line or subsequent therapy, the ORR was 28.07%, and the DCR was 94.15%.

### 3.3 Prognostic Assessment

As shown in Table 3, through Cox univariate regression model and Cox multivariate regression model analysis, factors such as age, BCLC staging, maximum tumor diameter, and presence of extra-pulmonary metastasis were found to be significantly correlated with prognosis. This suggests that patients over 60 years of age, in BCLC stage C, with a maximum tumor diameter greater than 10 cm, or with extra-pulmonary metastasis have a poorer prognosis (p<0.001).

### 3.4 Adverse Events

During the treatment, no patients experienced complications related to catheter placement. Other adverse events are shown in Table 4. Common adverse events included post-treatment fever and abdominal pain with IPCGOR combined with IL-2 treatment; increased liver enzymes, hypoalbuminemia, and hand-foot syndrome, among others. In terms of grade ≥3 adverse reactions, those occurring at a rate exceeding 10% were leukopenia (11.52%), thrombocytopenia (17.2%), and hand-foot syndrome (15.65%). A total of 448 patients (97.39%) experienced at least one adverse reaction of any degree, and 125 patients (27.82%) experienced adverse reactions of grade ≥3. After symptomatic treatments such as pain relief, leukocyte boosting, liver protection, blood pressure reduction, and reducing the dosage of targeted drugs, the adverse reactions improved. There were no treatment-related deaths.

## 4. Discussion

This study demonstrates that the IPCGOR regimen combined with IL-2 achieves favorable clinical efficacy with a tolerable profile of adverse reactions in patients with advanced NSCLC. Typically, patients with advanced NSCLC face poor clinical outcomes and limited survival due to the loss of surgical opportunities, evidenced by a mere 14.1% five-year survival rate 26-27. However, the IPCGOR combined with IL-2 offers new therapeutic hope, significantly reducing mortality and disease progression risks. The local treatment effects of IPCGOR, which include lesion destruction and tumor cell necrosis, release numerous tumor-associated antigens, thus amplifying the immune response 28-29. Concurrently, IL-2 administration appears to potentiate this immune response, enhancing treatment efficacy 30-31.IL-2 plays a pivotal role in modulating immune responses, crucially influencing T cell expansion, multiplication, and persistence, essential for combating oncogenic cells. In IPCGOR and IL-2 therapy, IL-2 acts as a potent immune stimulant, promoting the proliferation and activation of NK cells and CTLs, key to targeting and destroying tumor cells 32-33. The synergy between IL-2's immune amplification and IPCGOR's direct cytotoxicity results in a robust and comprehensive anti-cancer action.In terms of adverse events, our study observed a spectrum of reactions consistent with both chemotherapy and immunotherapy. The most common adverse events included chemotherapy-induced nausea, fatigue, and hematological abnormalities, as well as immunotherapy-related skin reactions and mild flu-like symptoms. Notably, these adverse events were generally manageable and less severe compared to those reported in patients undergoing more traditional chemotherapy regimens, such as platinum-based treatments, which often result in more significant hematological toxicities and gastrointestinal disturbances 34-35. The safety profile of IPCGOR combined with IL-2, therefore, appears comparatively favorable, particularly when considering the enhanced quality of life and reduced hospitalization duration it offers. Comparing the adverse event profile of IPCGOR and IL-2 with standard treatments, it is evident that while some side effects overlap, the intensity and frequency of severe reactions are markedly reduced in our study. This suggests that the combination of localized chemotherapy with systemic immunological enhancement not only improves clinical outcomes but also presents a safer alternative to more aggressive conventional therapies. Such insights underscore the potential of this integrative approach in treating advanced NSCLC, warranting further investigation in larger, randomized studies.

During our study, patient-reported experiences revealed a significant improvement in quality of life, a testament to the IPCGOR combined with IL-2 regimen's efficacy. This improvement was primarily attributed to the reduced treatment duration and the lesser severity of adverse reactions, particularly when compared to conventional chemotherapy regimens. The shorter infusion time and decreased frequency of hospital visits inherent in this treatment modality were highly valued by patients, as they substantially minimized disruption to daily routines and alleviated the psychological burden often associated with prolonged hospitalizations. Additionally, the manageable side-effect profile and comprehensive patient education facilitated a greater sense of empowerment and active participation in the treatment process. These observations are crucial, as they align with the emerging paradigm shift in NSCLC treatment, where patient-centric care is becoming increasingly important. Modern oncological research and practice are progressively acknowledging the significance of enhancing not just the quantity but the quality of life in cancer care. In this context, our study's findings contribute to the broader landscape of NSCLC research by offering evidence that supports a more humane and holistic approach to treatment. The IPCGOR combined with IL-2 regimen not only shows promising clinical benefits but also positively influences patients' lived experiences during their cancer journey. This aspect is particularly vital in the holistic management of advanced NSCLC, as it aligns with the evolving trends in oncology that prioritize patient well-being alongside clinical efficacy. Our study thus adds a valuable dimension to NSCLC treatment options, suggesting that this regimen could be a viable alternative to more traditional, and often more taxing, chemotherapy protocols. The implications of our findings extend beyond the immediate clinical outcomes, potentially influencing future research directions and clinical practices. By highlighting the importance of patient experience and quality of life, this study encourages further exploration into treatments that are not only clinically effective but also patient-friendly, ultimately aiming to transform the standard of care in NSCLC treatment.

The IPCGOR combined with IL-2 treatment delivers chemotherapeutic drugs directly to the patient's thoracic cavity through a catheter, directly killing NSCLC cells through toxic reactions and activating immunity through IL-2. This not only reduces the myelosuppression caused by IPCGOR but also activates immune cells for an anti-tumor effect. Currently, the IPCGOR combined with IL-2 treatment regimen has shown excellent efficacy. Propper etal's study 36 indicates that IPCGOR combined with IL-2 significantly improves PFS, OS, and ORR in patients with advanced lung cancer compared to the IPCGOR regimen alone,consistent with this study. This study shown that the mPFS of the IPCGOR combined with IL-2 regimen was 8.5 months, MOS was 12.5 months, and ORR was 58.3%, underscoring the significant implications of promoting IPCGOR combined with IL-2 treatment in progressive lung cancer, further confirming the effectiveness and safety of the IPCGOR combined with IL-2 regimen.

Currently, most studies on chemotherapy drug intrathoracic perfusion in NSCLC are based on the IIOT perfusion scheme 37-41. The IIOT scheme requires about 60 hours of perfusion, during which patients need to lie flat throughout, posing risks of catheter dislodgement, blockage, and infection. Therefore, modifying the chemotherapy regimen and shortening the treatment time have become urgent clinical issues to address 42-45. Gemcitabine inhibits the activity of thymidylate synthase, thereby suppressing intracellular DNA synthesis 46-48. With its long half-life, it can persist in tumor cells for an extended period to achieve an anti-tumor purpose 49-50. Moreover, the combination of gemcitabine and oxaliplatin can have a synergistic effect. The IPCGOR combined with IL-2 chemotherapy scheme requires only 4 hours of perfusion, significantly shortening the treatment time and improving patient comfort. This study shows that the ORR of IPCGOR combined with IL-2 treatment for advanced NSCLC is 67.4%, and the DCR is 97.4%, with mPFS of 8.5 months, respectively. The treatment-related adverse reactions in both groups are similar, mainly including elevated transaminases, decreased white blood cells and platelets, nausea, and vomiting. Therefore, with similar efficacy between the two regimens, the RALOX scheme reduces the perfusion time to 5 hours, decreasing the patient's bed rest time and reducing the incidence of catheter-related adverse events.

The strength of this study is its provision of robust evidence for the effectiveness of IPCGOR combined with IL-2 in treating advanced NSCLC, demonstrated through a large-sample clinical study conducted at a single center. This research confirms the feasibility of the IPCGOR combined with IL-2 treatment regimen for advanced NSCLC, adding valuable insights to existing literature. However, it is important to acknowledge several limitations inherent in our study's design. Being a single-center, retrospective study, it may have inherent biases, including selection bias and information bias. Selection bias could arise from the non-randomized selection of participants, potentially leading to a sample that may not be fully representative of the broader NSCLC patient population. Information bias may result from the retrospective collection of data, where inconsistencies in medical records or recall inaccuracies could affect the reliability of our findings. The retrospective nature of our study limits our ability to establish causality between the treatment and observed outcomes. Our conclusions are primarily based on observed associations, which might be influenced by confounding variables not accounted for in the study design. In future study, our team intends to address these limitations by conducting prospective studies. Such studies would allow for a more controlled environment to minimize biases and enable a more rigorous analysis of causality. This approach will provide more reliable and generalizable evidence for the efficacy of IPCGOR combined with IL-2 in treating advanced NSCLC. We recognize the need for multicenter trials to enhance the generalizability of our results. Collaborating with multiple centers would help in accruing a more diverse patient population, thereby offering a broader understanding of the treatment's effectiveness across different demographic and clinical settings.

## 5. Conclusion

The present study provides valuable insights into the efficacy and safety of the IPCGOR regimen combined with IL-2 for treating advanced NSCLC, underscoring its potential as a viable therapeutic option. We observed an Objective Response Rate (ORR) of 67.4% and a Disease Control Rate (DCR) of 97.4%, coupled with a median Progression-Free Survival (PFS) of 8.5 months and a median Overall Survival (OS) of 12.5 months. This regimen exhibited a tolerable adverse reaction profile, with no fatalities directly related to the treatment. The study also highlighted the significance of patient-specific factors, like age, BCLC staging, tumor diameter, and extra-pulmonary metastasis, in determining the prognosis, emphasizing the need for personalized treatment approaches. Our study opens several avenues for further research to build upon these findings. Given the retrospective nature of our research, there is a compelling need for prospective studies or randomized controlled trials to validate and extend our results. Such studies would offer more rigorous evidence regarding the efficacy and safety of the IPCGOR and IL-2 regimen, potentially confirming its suitability as a standard treatment option. Moreover, examining this treatment in diverse patient populations, including those with different stages of NSCLC or various comorbid conditions, would provide valuable insights into its applicability and effectiveness in broader clinical settings. These future investigations could also explore long-term outcomes, patient quality of life, and the economic aspects of the treatment, contributing to a more comprehensive understanding of its role in NSCLC management.

## Figures and Tables

**Table 1:** Baseline information on the study population

Variables	Number (%)/x±s/M
**Sex**	
**Male**	409 (88.91%)
**Female**	51 (11.09%)
**Age (Year)**	51.3±9.8
**BCLC staging**	
**B**	33 (7.17%)
**C**	427 (92.83%)
**Extrapulmonary metastasis**	
**No**	241 (52.39%)
**Yes**	219 (47.61%)
**Maximum tumour diameter (cm)**	12.8±2.6
**AFP (ng/mL)**	
**<400**	121 (26.30%)
**≥400**	339 (73.70%)
**ALT (U/L)**	41.0 (17.8,149.2)
**AST (U/L)**	49.0 (21.5, 219.3)
**Total bilirubin (μmol/L)**	14.4 (6.1,128.5)
**Albumin (g/L)**	35.4±3.7
**Prothrombin time (s)**	12.0±0.8
**Blood platelet (10^9^/L)**	191.2±92.9
**C-reactive protein (mg/L)**	15.9 (1.3,165.2)

**Table 2:** Evaluation of the efficacy of IPCGOR in combination with IL-2 in the treatment of NSCLC

Assessment of efficacy	n (%)
CR	0
PR	310 (67.4%)
SD	138 (30.0%)
PD	12 (2.6%)
ORR	67.4%
DCR	97.4%

**Table 3:** Analysing patients' post-treatment progression-free survival time by Cox regression

Variables	One-factor Cox proportional risk regression model analysis		Multi-factor Cox proportional risk regression model analysis	
	HR value (95%CI)	p value	HR value (95%CI)	*p* value
**Sex**				
**Male**	1		/	
**Female**	1.51 (1.28-1.70)	0.825	/	/
**Age**				
**≥60**	1		1	
**<60**	0.87 (0.72-0.98)	<0.001	0.84 (0.76-0.89)	<0.001
**BCLC staging**				
**B**	1		1	
**C**	0.62 (0.53-0.81)	<0.001	0.59 (0.47-0.73)	<0.001
**Extrapulmonary metastasis**				
**No**	1		1	
**Yes**	0.77 (0.53-0.91)	<0.001	0.76 (0.55-0.87)	<0.001
**Vascular invasion**				
**No**	1		/	
**Yes**	0.63 (0.49-0.71)	0.238	/	/
**Maximum tumour diameter**				
**<10cm**	1		1	
**≥10cm**	0.65 (0.54-0.75)	<0.001	0.63 (0.57-0.74)	<0.001
**ALB**				
**<35g/L**	1		/	
**≥35g/L**	1.68 (1.40-1.83)	0.392	/	/
**PT**				
**<12s**	1		/	
**≥12s**	1.90 (1.71-1.98	0.408	/	/
**PLT**				
**≥200×109/L**	1		/	
**<200×109/L**	1.35 (1.29-1.50)	0.670	/	/
**CRP**				
**<16mg/L**	1		/	
≥16mg/L	0.79 (0.68-0.85)	0.785	/	/
AFP				
<400ng/mL	1		/	
≥400ng/mL	1.47 (1.39-1.56)	0.836	/	/

**Table 4:** Adverse event statistics during treatment

Adverse reaction	<3 level adverse reaction	≥3 level adverse reaction
High temperature	241 (52.39%)	4 (0.86%)
Stomach pain	218 (47.39%)	2 (0.43%)
Nausea	96 (20.87%)	0
Vomiting	116 (25.22%)	0
Diarrhea	36 (7.82%)	1 (0.22%)
Leucopenia	232 (50.43%)	53 (11.52%)
Thrombocytopenia	265 (57.60%)	79 (17.2%)
Elevated liver enzymes	355 (77.17%)	4 (0.87%)
Low albumin	386 (83.91%)	0
Elevated total bilirubin	116 (25.21%)	0
Proteinuria	89 (19.34%)	0
Hypertensive	172 (37.39%)	0
Gastrointestinal bleeding	23 (5.00%)	0
Hypothyroidism	29 (6.30%)	0
Hand-to-foot syndrome	69 (15.00%)	87 (18.91%)
Skin capillary hyperplasia	9 (1.95%)	0
Rashes	59 (12.82%)	3 (0.65%)
Hoarse	8 (1.73%)	0
